# Observations on the Treatment of Hysteria

**Published:** 1819-10

**Authors:** J. Mahon


FOR THE LONDON MEDICAL AND PHYSICAL JOURNAL.

### *Observations on the Treatment of Hysteria.* By J. MAHON, M.D.

MY observations on amenorrhœa, inserted in your Journal for November last, were accompanied with an intimation that I should resume the subject of uterine disorders on another occasion; and this I have neglected to do only from an intention of treating the diseases especially incident to women, in an extensive, and indeed a general, manner. I am preparing a series of essays on them, which, when completed, I will transmit to you: but, as some considerable period of time may elapse before they appear, I am induced now to make some remarks on the treatment of the hysterical paroxysm, from a belief that serious injury is daily effected by the means in vulgar use. I should here observe, that it is not my intention at present to treat of the pathology of hysteria any further than may be necessary to explain my reasons for inculcating the use of the measures I shall describe.

Hysteria most frequently affects young females, or those living in a state of celibacy, who have been educated in a luxurious manner as regards both diet and bodily exercise, who are daily subject to the emotions consequent on restrained natural passions, and whose mental functions have been inordinately developed at the expense of those of the rest of the economy. It is, like mania from gastric disorder, peculiar to civilized life; and shows, like that affection, the results of an inordinate susceptibility of the brain to impressions from the rest of the system. Hysteria generally affects those of full habits: it, at least, generally first occurs in that state of the system; but, like all the consequences of impressions on the sensorium, it is very likely to happen subsequently from what is commonly, though vaguely, termed *habit*, in a totally different state of the constitution, and from very trivial causes. It does sometimes primarily occur in a spare and weak state of the constitution; but, in this case, it is always from the influence of inordinate passionate emotions; it never happens as the consequence of mere debility of the system: females dying of marasmus from hæmorrhage are hardly ever affected with it. But, although it depends on a state of inordinate excitement, the hysterical paroxysm, when it affects persons in a state of debility, may sometimes be relieved by stimulants applied to the stomach; probably by producing a determination to the abdominal viscera, and thus removing the excitement of the brain; and such remedies have therefore been commonly resorted to in every kind of hysterical affection, by a great proportion of medical practitioners. But, if they would contemplate a young female in this state, who had previously been in high health, with a face now tumid and suffused with blood, and a full and throbbing pulse; if they would contemplate this scene with a mind unbiassed by the farrago of confused ideas attached to the terms convulsions, antispasmodics, &c. and consider attentively the effects of stimulating medicines, they must be convinced that such medicines prove highly injurious; and yet to what an extent are æther, volatile alkali, assafœtida, &c. commonly administered on those occasions. As soon as the violence of the fit has subsided, and the patient has become able to swallow, the opportunity is seized to pour down some highly stimulating liquid; and a return of convulsions is generally produced. A fit of hysteria, in a full-blooded female, is, under such medical treatment, rarely of less than four, five, or six, hours' duration; and is followed by severe head-ach and disorder of the stomach for several days, and the disposition to a recurrence of the disorder much promoted: whilst, by an opposite mode of treatment, it may, in a great portion of cases, be almost instantaneously removed.

I was, early in my practical career, led to relinquish the stimulating plan of treating hysteria in young females; but it was not until the case I am about to relate occurred to me, that I was led to pursue the method I shall presently describe.

I was called to visit a patient, an unmarried woman, 22 years of age, and of a spare habit of body, who had been about an hour in a severe hysterical paroxysm. I learned from the attendants, that she had complained of pain about the loins and hypogastric regions for two or three weeks previously; that menstruation had been effected irregularly, and somewhat profusely, for four or five months past; and that, within the last eight days, she had before experienced three severe fits of hysteria, each of which continued four or five hours. On passing my hand over the abdomen, with a view of ascertaining if there were any tension or swelling in that part, I was somewhat surprised to feel a violent throbbing about the epigastrium, in the situation of the cœliac artery. This furnished an indication, which I immediately followed, and abstracted twelve ounces of blood. In five minutes the patient became perfectly sensible, and she experienced no return of the convulsions. Blood-letting and purging were afterwards employed.

Since then I have always examined the abdomen in patients in a fit of hysteria, and have very frequently, in thin and young persons, detected a similar pulsation of what I suppose to have been the cœliac artery: it is almost needless for me to say, that I have since then but rarely employed stimulating internal medicines in the treatment of this disorder.

The measures, then, on which I have placed most reliance, have been bleeding, cold air, cold drinks, and especially cold applications to the abdomen; and I have sometimes ordered one of the attendants to dash a vessel of cold water over the lower part of the belly and the thighs. The effect of the cold application to the region of the pubes, is often almost surprising: the patient utters a scream, starts up, and looks about with astonishment, like a person suddenly roused from sleep, enquiring what is the matter, and the cause of so many persons being about her.

In cases in which I have not employed blood-letting, and where the cold application to the pubes has produced only temporary relief, I have given an emetic of ipecacuanha, followed by nauseating doses of the same medicine, with the most advantageous results. On this point I find I am supported by the opinions of Dr. Smith, of New York, given in your Journal for October last. I should here observe, that, in this, and in other cases where the functions of the brain are much disturbed, ipecacuanha, not tartar emetic, sulphate of zinc, &c. should be employed: it is common to find the latter substances used, from the difficulty with which vomiting is then excited; but this practice is highly deleterious, causing severe injury to the stomach, from the large quantities of those substances often required: of this I have witnessed several instances. There is not this danger attendant on the use of ipecacuanha; and, where the system is so much disordered that this will not produce vomiting, I would strongly advise that the attempt to cause it should be desisted from. I have but rarely employed opium in the treatment of hysteria, and only after the paroxysm has subsided, with the view of procuring sleep and quieting the system; and, even with this intention, I have almost confined its use to patients of a weak and spare habit of body, or when the affection is habitual, or has been immediately caused by some sudden mortal emotion.

The measures I have described have not only been found to be particularly efficacious in removing the paroxysm of hysteria; there is this advantage they also possess over the usual mode of treatment,—they are not followed by the head-ach and disorder of the stomach so commonly consequent on the use of violent stimulants, and they tend to prevent the recurrence of the disease. On the latter point I shall not now dwell. My object has been to prevent the use of the common deleterious modes of treating the hysterical paroxysm, by proposing that which I have myself found to be remarkably beneficial; and which, I trust, will be equally so in the hands of other practitioners, if employed with common judgment. My opinions on the pathology, and remarks on the general management, of this disorder, will be comprised in the essays to which I have alluded.

